# Gastrointestinal microbiome and gluten in celiac disease

**DOI:** 10.1080/07853890.2021.1990392

**Published:** 2021-10-14

**Authors:** Xingxing Wu, Lin Qian, Kexin Liu, Jing Wu, Zhaowei Shan

**Affiliations:** a Affiliated Hospital of Nanjing University of Chinese Medicine, Nanjing, China; b Institute of Chinese Medicine, Nanjing Drum Tower Hospital, Nanjing University, Drum Tower Clinical Medicine College of Nanjing University of Chinese Medicine, Nanjing, China

**Keywords:** Gluten, gastrointestinal microbiome, coeliac disease, gluten free diet, probiotics

## Abstract

Coeliac disease (CD), also known as gluten sensitive enteropathy, is an autoimmune intestinal disease induced by gluten in genetically susceptible individuals. Gluten is a common ingredient in daily diet and is one of the main environmental factors to induce coeliac disease. Adhering to gluten free diet (GFD) is an effective method for treating CD. Microbiota plays an extremely important role in maintaining human health, and diet is the main factor to regulate the composition and function of gut microbiota. Recent studies have shown that gluten metabolism is closely related to gastrointestinal tract (GIT) microbiota. With the increasing prevalence of coeliac disease, there is a need for alternative treatments to GFD. In this review, biological medication of gluten, relationship between gluten and gut microflora, effect of GFD on GIT microflora, and effect of probiotics on CD were reviewed. By analysing the research progress on relationship between gluten and gastrointestinal microbiome in coeliac disease, this review tried to explore the prospective and potential mechanism of microecological agents in treating coeliac disease.

## 1. Introduction

Coeliac disease is an autoimmune disorder that occurs in genetically predisposed individuals, including adults and children, who develop an immune reaction to gluten. Even though this disease primarily affects the small intestine, its clinical manifestations are broad, with both intestinal and extra-intestinal symptoms. There are multi-factors might affect this disease, such as environmental, genetic factors and immune imbalance [1]. The main clinical presentations include intestinal symptoms, such as diarrhoea, abdominal distension, abdominal pain; and extra-intestinal symptoms, such as anaemia, dermatitis herpetiformis, osteopenia and peripheral neuropathy. CD patients carry specific susceptibility genes (HLA-DQ2, HLA-DQ8), but their existence is not enough to cause the occurrence of CD, which requires the participation of environmental factors-gluten [2]. As the consumption of gluten-containing food increases, the incidence of related autoimmune diseases has gradually increased (such as CD, wheat allergic diseases) [3]. In genetically susceptible individuals, gluten is one of the necessary conditions for inducing CD. Gluten is a kind of protein mainly existing in wheat, barley and rye, accounting for 80%–85% of the total protein in wheat [4]. It is a protein mixture composed of hundreds of monomers, oligomers and polymers, mainly including gliadin and glutenin. Among them, the main antigen protein causing CD is gliadin which is rich in glutamine and proline, and cannot be digested by human digestive enzymes and brush border peptidase [5–6]. Proline-rich peptides are protected from proteolysis by gastric, pancreatic and intestinal brush border membrane enzymes, so they have an opportunity to build up to high concentrations in the small intestine. However, oral bacteria that secrete gliadin (gluten) degrading enzymes had been identified. Their most active gliadin-cleaving enzymes had also been identified and purified [7–8]. Apart from interesting biological findings, these bacteria and enzymes may lead to novel and effective strategies to detoxify immunogenic gluten peptides prior to their reaching the proximal small intestine. Part of the gluten is hydrolysed by oral microbial proteases in the oral cavity, thereby reducing its immunotoxicity. However, most gluten is hydrolysed by pepsin into high molecular weight peptides in the stomach. The peptides that enter the small intestine from the stomach are not easily degraded due to their rich proline. They stay in the intestine for a long time and increase the probability of triggering immune response. A large number of immunogenic polymer peptides gathered in the intestinal lumen, mainly including immunodominant peptides (such as P57-P89 peptide and 33 peptide in α-gliadin) and non-immune dominant peptides (such as P31∼P43), triggered the adaptive immune response mediated by CD4 + Th1 cells and the innate immune response mediated by intraepithelial lymphocytes respectively, and leaded to infiltration of intestinal epithelial inflammatory cells, villus atrophy and crypt hyperplasia [9]. Thus, it will lead to the destruction of intestinal epithelial cells and the increase of intestinal permeability, resulting in diarrhoea, abdominal distention, abdominal pain, emaciation, dermatitis herpetiformis and other clinical symptoms. Although the gluten-free diet can significantly improve the clinical symptoms of patients with CD, gluten-free diet is expensive and has very few products. In order to have a good quality of life, patients have to adhere to the gluten-free diet. It not only brings a financial burden to society and patients themselves, but also brings social and psychological impact to patients [10]. In addition to gluten, the microbiota dysbiosis of the digestive tract flora may be another environmental factor that triggers CD. Studies have confirmed that patients with CD have disorders of the digestive tract flora. The abundance and diversity of beneficial commensals have decreased, while, pathobionts have increased. Coeliac disease is associated with intestinal dysbiosis characterized by increases in pathobionts virulence features [11–12]. Research also shown that, diet has a great influence on the composition and function of intestinal flora, and gluten can affect the stability of intestinal flora. Therefore, this review will mainly focus on the relationship between gluten and oral flora, intestinal flora in coeliac disease. It will also pay special attention to analyse perspectives and trends of probiotics in coeliac disease treatment.

## 2. Gluten and oral flora

### 2.1. Oral flora

So far, it has been found that there are more than 1,000 kinds of bacteria in the oral cavity inhabiting saliva, teeth, gingiva and other different parts. There are about 10^11^ bacteria per gram of dental plaque and 10^8^ bacteria per millilitre of saliva, so the oral cavity becomes the second place where microorganisms are densely colonized in digestive tract [13–14]. The latest research shows that the oral symbiotic flora can increase the immune function of the oral mucosa to prevent the invasion of pathogenic microorganisms. For example, oral microbiota genera *Veillonella* and *Streptococcus* promote the production of anti-microbial peptides and the secretion of inflammatory cytokines, increasing the epithelial barrier function and thickness characteristic of oral mucosa [15]. However, other studies have found that some pathogenic bacteria in oral microflora are not only related to oral diseases such as dental caries, periodontitis, and oral ulcers [16], but also related to infective endocarditis [17] and coronary atherosclerosis [18], pneumonia [19], obesity [20], intestinal diseases, etc. The flora in the oral cavity of healthy humans can be transported in large quantities to the distal end of the digestive tract, yet the translocation of oral species to the intestine is considered a rare aberrant event, and a hallmark of disease [21]. Certain oral microorganisms may induce intestinal diseases in genetically susceptible individuals. For example, Atarashi et al. found that oral *Klebsiella* colonises the colon and induces Th1 cell differentiation to elicit a severe intestinal mucosal inflammation [22].

### 2.2. Oral flora reduces the immunogenicity of gliadin

At present time, most studies are confined largely to explore the relationship between microflora and intestinal diseases. However, the oral cavity is the first digestive organ that comes into contact with food and is correlated to digestive system diseases directly. Therefore, on the basis of duodenal flora and colonic flora studies, the salivary flora should be further analysed to improve description of the digestive tract flora characteristics [23]. Patients with CD have oral flora dysbiosis. There are microbial flora in the oral cavity, which are related to the metabolism of gluten in CD (Table 1). Although the food containing gluten stays in the oral cavity for a short time, the number and types of flora in the saliva are significantly greater than those colonized in the stomach and duodenum. The effect of oral flora on digesting gluten should not be ignored. Researchers have found that the initial metabolism of gliadin in the oral cavity may be related to the genus of *Rothia*, *Actinomyces*, *Neisseria*, and *Streptococcus* that colonized the oral cavity [24]. Compared with healthy people, the saliva of patients with CD is rich in bacteria that could degrade gluten, and the degradation rate of gluten is higher. The significant increase of *Lactobacillus* species may be one of the reasons [23]. The protease-resistant highly immunogenic 33-mer α-gliadin peptide could be completely degraded by dental plaque bacteria to reduce immunogenicity [23,25]. However, there were studies on the contrary standpoint, reported that oral microbial enzymes degrade part of gluten, which in turn increases immunogenic small molecule peptides epitopes and further induces intestinal inflammation [23].

**Table 1.** The relationship between Oral flora and gluten in CD.

	Substrate types	Degradability	Outcome
Salivary flora [23]	Gluten	The degradation rate of gluten is higher than healthy people	unspecified
*Rothia, Actinomyces, Neisseria, and Streptococcus* [24]	Gliadin	unspecified	unspecified
Dental plaque bacteria [23,25]	Immunogenic 33-mer α-gliadin peptide	Complete degradation	Reduce immunogenicity
Oral microbial enzymes [23]	Gluten	Partial degradation	Induce immunogenicity

## 3. Gluten and intestinal flora

### 3.1. Intestinal flora

As we mentioned before, the oral cavity is the second place where microorganisms are densely colonized in digestive tract. However, the gut is the most densely colonized place of microflora in digestive tract. A refined estimate showed that microflora in one human body were in a ratio of 1.3:1 to human cells. It was estimated that more than 1,000 kinds of microorganisms live in the gut, the gut microbiome of healthy people mainly includes *Firmicutes*, *Bacteroides*, *Proteobacteria*, *Actinomycetes*. And some researchers estimated that there were thousands of bacterial species in the gastrointestinal tract [26–27]. According to the interaction with the host, the intestinal flora is divided into three categories: probiotics (such as *Lactobacillus*, *Bifidobacterium*, etc.), pathobionts (such as *Clostridium*, *Enterococcus faecalis*, etc.) and opportunistic pathogens. The intestinal flora of healthy people can protect and maintain the intestinal barrier function, promote the metabolism and absorption of nutrients, regulate immunity, anti-aging, prevent cancer and suppress cancer, etc [28]. There is a mutually symbiotic relationship between the flora and the host. The host provides nutrients and the microenvironment for the flora. The flora helps to maintain human intestinal homeostasis by participating in a series of physiological functions of the host. A large number of studies have shown that once the balance between intestinal microflora and the human body is broken, it will lead to multiple systemic diseases, such as obesity, diabetes, atherosclerosis, irritable bowel syndrome, inflammatory bowel disease, and coeliac disease through bile acid metabolism, brain-gut axis, intestinal barrier, and immune system and so on [29].

### 3.2. Gliadin directly induces intestinal flora dysbiosis

For the coeliac disease patients, the balance between intestinal microflora and the human body could be broken by the gliadin. From the mouth and stomach, large quantity of undegraded gliadin is being pushed into the small intestine and large intestine, provides abundant substrates for different bacteria in the intestinal cavity, thereby promotes the reproduction of gliadin-degrading bacteria and breaks the steady state of intestinal flora [30–31]. At present time, the composition and structure of the small intestinal flora are mainly evaluated by detecting the abundance and diversity of the duodenal flora. D’Argenio et al. tested the duodenal mucosal flora of patients with active CD and found that the abundance of *Proteobacteria* increased, while the abundance of *Firmicutes* and *Actinobacteria* decreased. Compared with GFD patients and healthy individuals, members of the *Neisseria* genus (*Betaproteobacteria* class) were significantly more abundant in active CD patients. *Neisseria flavescens* was the most abundant *Neisseria* species in active CD duodenum [32]. Sanchez E et al. found that compared with children with GFD and healthy children, the duodenal-mucosal bacteria of children with active CD (normal gluten-containing food diet) has increased abundance of *Proteobacteria* and decreased abundance of *Firmicutes* at the phylum level; the abundance of *Enterobacteriaceae* and *Staphylococcaceae* increased, and *Streptococcaceae* decreased at family level [33]. In the CD animal experiment, it was also found that the intestinal flora was imbalanced. For example, comparing the gluten-sensitive (GS) macaques with healthy macaques, it was found that the alpha diversity (Shannon diversity index) and abundance of the faecal flora of the GS macaques were reduced, and it was found that, two of the top eight families, *Streptococcaceae* and *Lactobacillaceae*, were enriched in GS macaques [34].

### 3.3. Intestinal flora promotes the hydrolysis of gliadin

The human body lacks proteases, which are able to completely digest gluten. The role of intestinal flora in the process of such protein metabolism cannot be ignored. The undegraded gliadin is transported from the small intestine to the large intestine. Once it enters the large intestine, it is in close contact with a large number of microorganisms in the gut. Due to the diversity of bacterial genes in large intestine and their different biochemical pathways from the human body, it makes certain intestinal microorganisms have the ability to metabolize gliadin [35]. Researchers have found that there are flora related to the metabolism of gliadin in the human intestine (such as the genera *Lactobacillus*, *Streptococcus*, *Staphylococcus*, *Clostridium*, *Bifidobacterium*) [36]. These microorganisms not only exist in the large intestine, but also in the small intestine to metabolize gluten. Camino et al. showed that, compared with the healthy group, the duodenal mucosal flora of CD mice on a gluten-containing diet had a higher proteolytic activity against gluten (“glutenasic” activity), and it is related to the abundance of *Proteobacteria* (including *Pseudomonas*) [37]. Herrán et al. studied the small intestinal flora that decomposes gliadin in healthy people and patients with CD, and isolated 114 bacterial strains belonging to 32 different species from the duodenal mucosa, of which, 85 strains were able to grow in a medium containing gluten as the sole nitrogen source. 31 strains showed extracellular proteolytic activity against gluten protein and 27 strains showed peptidolytic activity towards the 33-mer peptide, an immunogenic peptide for coeliac disease patients [38].

### 3.4. Gliadin combined with intestinal flora induces intestinal inflammation

Obviously, researchers cannot determine that intestinal flora dysbiosis is the result of coeliac disease or an environmental factor for CD, or both. Conventionally, gliadin was considered to activate innate immunity and adaptive immunity, and activate intestinal inflammation by inducing the production of cytokines and chemokines. In particular, the gliadin is deamidated by tissue transglutaminase in the lamina propria of the small intestine, and binds to HLA class II DQ2/8 molecules of antigen-presenting cells, activates T cells, macrophages and dendritic cells, and secretes inflammatory cytokines. It follows the activation of the adaptive immune response through the production of anti-endomysium, antigliadin, and anti-transglutaminase antibodies by B cells that increase intestinal permeability [39]. In addition to gliadin, intestinal microflora also play an indispensable role in inducing inflammation in the intestinal mucosa of patients with CD. As we all know, immune factors are one of the causes for CD, and adaptive immunity plays an important role in the pathogenesis of CD. Studies have found that the intestinal flora is closely related to adaptive immunity, and intestinal microflora have important regulatory effects on the two embranchments of host adaptive immunity, B cells and T cells. The intestinal flora can promote the production of IgA in the intestine by regulating the B cells response; it can also maintain the balance between intestinal inflammation and immune tolerance by inducing the differentiation of intestinal Th17 and Treg cells [40].

Gliadin evokes intestinal barrier dysfunction, which leads to the excessive growth and translocation of intestinal pathogenic bacteria, resulting in intestinal microecological imbalance; The microecological imbalance activates the immune inflammatory response by regulating the B cell and T cell. Inflammatory factors can further increase the permeability of the intestinal mucosa by destroying the intestinal epithelial cells and aggravate coeliac disease [41]. The CD intestinal mucosal immune response may directly destroy the biological barrier, thereby affecting the microbial homeostasis. The imbalance of the flora, or dysbiosis acts as a pathogenic factor to be counteractive at CD, thus forming a vicious circle and continuing inflammation.

**Figure 1.**
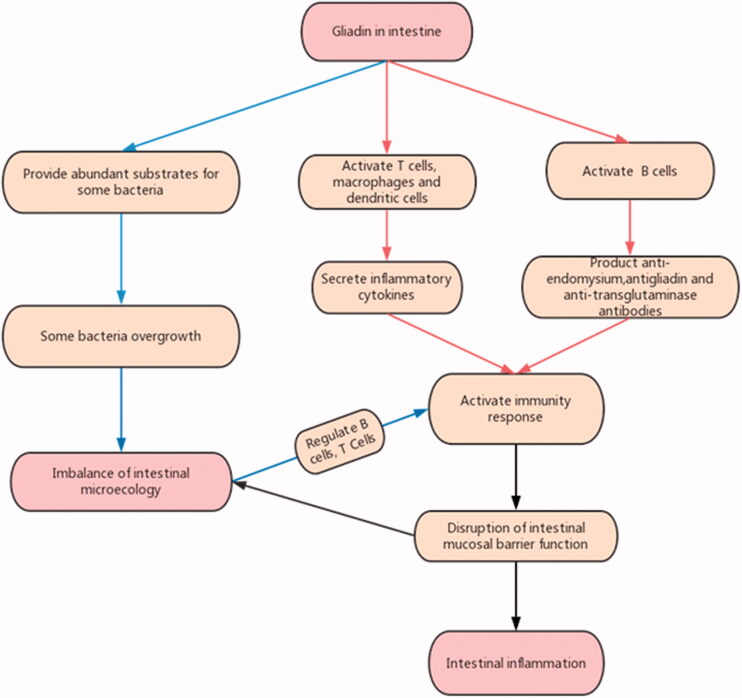
The relationship between Intestinal flora and Gluten in CD.

In CD, intestinal flora and gluten have a complex relationship (Figure 1). There are two essential different situations, one is the imbalance of intestinal microecology caused by coeliac disease, the other is abnormal intestinal flora, which is a co-factor of gliadin in inducing coeliac disease. In CD patients, the abundance of *Firmicutes* and Actinobacteria decreased, while the abundance of *Proteobacteria* increased. The intestinal microflora might be sometimes the cause and sometimes the result, which needs analysis case by case. In the future, reasonable research methods can be designed to confirm it. With the development of research, the role of *lactic acid bacteria* can be further defined.

## 4. Gluten-free diet and digestive tract flora

CD patients have disorders of the digestive tract flora. However, has the digestive tract flora of CD patients improved significantly after GFD treatment? Studies have found that the digestive tract flora of CD patients who are treated with GFD is still in an imbalanced state. A study confirmed that, compared with the same number of healthy children, *Bacteroidetes* in saliva of children with GFD (*n* = 13) increased, *Actinobacteria* and *Streptococcus thermophilus* decreased [42]. Di Cagno et al. found that the duodenal mucosal flora of CD patients had not fully recovered after two years of GFD treatment. Although the abundance of the pathogenic bacteria declined, the abundance of the beneficial bacteria was still low [39,43]. Research by De Palma et al. showed that after healthy adults persisted in GFD, *Bifidobacterium*, *Lactobacillus*, and *Bifidobacterium longum counts* decreased, while the *Enterobacteriaceae* and *Escherichia coli* increased [44]. A similar study in CD children outlined differences in the microbiota composition before and after GFD treatment; mean Dice similarity index between coeliac individuals before and after GFD treatment was 63.9% ± 15.8%. There’s a loss of 36.1% of inter-individual similarity. This study also found that, *Bacteroides vulgatus* and *Escherichia coli* were detected more often in CD patients than in controls (Functional Dyspepsia), and a significant higher biodiversity in CD paediatric patients’ duodenal mucosa was shown [45]. GFD is an important factor affecting the composition of the intestinal flora. GFD not only fails to fully restore the digestive tract flora of CD patients, but also affects the homeostasis of the flora in healthy people (Table 2). A GFD diet clearly influences the abundance of several species, in particular those involved specifically in carbohydrate and starch metabolism such as family *Veillonellaceae* (class *Clostridia*) [46]. Diet is an important factor affecting the abundance, diversity and function of the flora. Under physiological conditions, dietary patterns and nutritional status have certain effects on the intestinal flora [47–48]. Among different dietary components, fibre has a positive effect on gut microflora and their related metabolites. Compared with standard diet, GFD contains less fibre [49–51]. Therefore, it can be preliminarily inferred that, GFD, which contains a small amount of fibre, is a factor leading to the dysbiosis of intestinal flora.

**Table 2.** The influence of GFD on gastrointestinal flora.

	CD with GFD	Healthy people with GFD
Francavilla et al. [42]	Decreased: *Bifidobacterium*, *Lactobacillus*, and *Bifidobacterium* *longum* Increased: *Actinobacteria* and *Streptococcus thermophilus*	–
Di Cagno et al. [43]	Decreased: pathogenic bacteria declined	–
De Palma et al. [44]	–	Decreased: *Bifidobacterium*, *Lactobacillus*, and *Bifidobacterium longum* Increased: *Enterobacteriaceae* and *Escherichia coli*
Schippa et al. [45]	Increased: *Bacteroides vulgatus* and *Escherichia coli*; biodiversity of flora	–
Bonder et al. [46]	–	Decreased: *Veillonellaceae*

## 5. The effect of probiotics on CD

There were not much studies about probiotics in affecting CD (Table 3). Olivares et al. found that *B. longum CECT 7347* could help improve the health status of CD patients who tend to show alterations in gut microbiota composition and a biased immune response even on a GFD [52]. A strict diet without gluten is the only effective way to treat CD for present time. Although long-term GFD can improve the symptoms of CD patients, there are still existing intestinal flora dysbiosis. At present, there are few studies on using probiotics as an intervention for CD patients on the basis of GFD. However, these limited research results still show that probiotics combined with GFD can restore the intestinal flora of CD patients. Studies have confirmed that the ratio of *Firmicutes* to *Bacteroides* and the abundance of *Actinobacteria* decrease in children with CD when compared with healthy controls. In children with GFD, oral probiotics containing two *Bifidobacterium* strains (B632 and BR03) were taken for 3 months. Compared with children with GFD who were not supplemented with probiotics, ratio of *Firmicutes* to *Bacteroides* and the abundance of Actinobacteria increased more than before. And it is basically similar to the faecal flora composition of healthy children [53]. Probiotic administration has clearly revealed a negative relationship between *Firmicutes* and pro-inflammatory TNF-a. Moreover, probiotic effect has exposed some new phyla, particularly *Synergistetes*, which negatively correlated to acetic acid and total SCFAs, suggesting a potential role in microbiome restoration [54]. A 6-week probiotic treatment is effective in improving the severity of IBS-type symptoms in CD patients on strict GFD, and is associated with a modification of gut microbiota, characterized by an increase of *Bifidobacteria* [55].

There also was study showed that, the probiotic formula when taken orally over the 12-week period did not significantly alter the microbiota of CD patients who were strictly adhere to GFD. The probiotic bacteria contained 450 billion viable lyophilized bacteria *Streptococcus thermophilus*, *Bifidobacterium breve*, *Bifidobacterium* *longum*, *Bifidobacterium infantis*, *Lactobacillus acidophilus*, *Lactobacillus plantarum*, *Lactobacillus paracasei*, and *Lactobacillus delbrueckii subsp. bulgaricus*. [56].

**Table 3.** The effect of probiotics on CD with GFD.

	Positive result	Negative result
GFD combined *B. longum CECT 7347* [52]	Improve the health status of CD patients	–
GFD combined probiotics (containing two *Bifidobacterium* strains) [53]	Restore intestinal flora basically	–
GFD combined probiotics [55]	Increase of *Bifidobacteria*; improve severity of IBS–type symptoms of CD patients	–
GFD combined probiotics [56]	–	No effect on intestinal flora

## 6. Summary and outlook

In summary, coeliac disease, gluten, and digestive tract microflora have complex interactions (Table 4). CD patients not only have intestinal flora dysbiosis, but also accompanied by oral microbial dysbiosis. The current research has not yet determined the exact microbial model of microflora and CD, and the causal relationship between the imbalance of the digestive tract flora and CD is still inconclusive. Gliadin and digestive tract flora are the environmental factors that induce CD, and there is a close relationship between the two factors. In the process of CD intestinal mucosal immune response, gliadin and flora play a synergistic effect, so that the intestinal immune response is continuously activated, causing clinical symptoms of CD. In addition, gluten-containing food provides abundant material energy for the digestive tract flora, which further leads to the imbalance of the flora. Some specific bacteria or some bacterial metabolites in the digestive tract can degrade gliadin even though mammals lack proteases to digest gliadin. On one hand, there is a causal relationship between gliadin and the imbalance of the flora. On the other hand, there is a synergistic relationship in the process of inducing CD intestinal immune response. In the future, we should conduct intensive research to clarify the common role of intestinal microecology and gluten in the pathogenesis of CD. Nevertheless, alterations of microbiota in CD subjects may not be considered exclusively as a consequence of the disease itself, but rather as a part of a complex relationship between many causative factors, including those of diet and psychological nature.

Persistence of symptoms in patients with CD who adhere to a GFD is common. Probiotics (especially *Bifidobacterium* and *Lactobacillus* related to gliadin metabolism) are expected to become an adjuvant preparation for CD patients and minimize the related adverse reactions caused by strict GFD. Probiotics may help to control symptoms in patients with CD adhering to a GFD, however, the data are limited and this could not be an absolute prediction. After all, previous research had shown that *Lactobacillaceae* were enriched in gluten sensitive animal models, so it cannot be ruled out the possibility that *lactobacilli* could not act as probiotics at some time. Future research studies involving high-quality clinical trials are needed to improve the quality of the evidence and to establish the optimal species, timing, and dosage of probiotics that may benefit patients with CD [57].

**Table 4.** Summary of changes in the digestive tract flora of patients with coeliac disease and healthy individuals with GFD.

	Increase			Decrease		
Site	A- CD	T-CD	H-GFD	Active CD,	T-CD	H-GFD
Oral	*Lactobacillus* species [23] (A-CD VS HC)	*Bacteroidetes* [42] (T-CD^1^ VS HC)	–	–	*Actinobacteria*, *Streptoco-ccus thermophilus* [42] (T-CD ^1^ VS HC)	–
Small intestine (Duodenal biopsy)	*Proteobacteria* [32,33], *Neisseria* genus [32], *Enterobacteriaceae* and *Staphylococcus* [33] CD VS T-CD ^1^, HC) *Bacteroides vulgatus* and *Escherichia coli* [45] (A-CD VS FD)	–	–	*Firmicutes* [32,33], *Actinomycetes* [32] *Streptococcaccae* [33] (A-CD VS T-CD^1^, HC)	–	–
Large intestine (faecal)	–	the ratio of *Firmicutes* to *Bacteroides*, *Actinomycetes* [53] (T-CD ^3^VS T-CD^2^), *Bifidobactea* [55] (T-CD^3^ VS T-CD^2^)	*Enterobacteriaceae* and *Escherichia coli* [44] (H-GFD VS HC)	the ratio of *Firmicutes* to *Bacteroides*, *Actinomycetes* [53] (A-CD VS HC)	–	*Bifidobacterium*, *Lactobacillus* and *Bifidobacterium longum* [44] (H-GFD VS HC)

A-CD: Active CD; T-CD: Treated Coeliac Disease (1: CD with GFD; 2:CD with GFD combined probiotics; 3: CD with GFD combined placebo); H-GFD: Healthy individuals with GFD; HC: Healthy controls; FD: Functional Dyspepsia.

## Author contributions

Conceptualisation, WU Xing xing, QIAN Lin and LIU Kexin,writing- original draft preparation, WU Jing and SHAN Zhao wei, editing review. All authors have read and agreed to the published version of the manuscript.
